# The Importance of Green Certification Labels/Badges in Online Hotel Booking Choice: A Conjoint Investigation of Consumers’ Preferences Pre- and Post-COVID-19

**DOI:** 10.1177/19389655231184474

**Published:** 2023-07-04

**Authors:** Guy Assaker, Peter O’Connor

**Affiliations:** 1 Lebanese American University, Byblos, Lebanon; 2 University of South Australia Business School, Adelaide, Australia

**Keywords:** green certification, online hotel selection, COVID-19, conjoint analysis

## Abstract

In light of travelers’ growing preference for sustainable hotel accommodation, this study investigated the relative importance of green certification labels/badges in online hotel selection/booking choice. A conjoint analysis was performed on seven online hotel attributes (including green certification labels/badges) in two specific scenarios (imagining they were booking in a pre- and post-COVID-19 setting) using 270 U.S. subjects surveyed in April 2020. The results revealed that green certification labels/badges do impact travelers’ online hotel booking choice, but not as much as cancellation policies, hotel rating, price, and location. Price in particular exerted a greater influence on travelers’ booking preferences in the post-COVID-19 scenario. Yet, a significant percentage of travelers (around 40% in both scenarios) were willing to pay more for a hotel with green certification. These results contribute to our theoretical and practical understanding of the factors that influence online hotel booking, as well as the power of green certification labels/badges in driving online hotel bookings in the pre-and post-COVID-19 contexts.

## Introduction

The last two decades have witnessed a change in travelers’ behavior in terms of how they select hotel accommodation and the factors they consider when shopping and booking a hotel stay (Toh et al., 2011; Verma & Chandra, 2018). On one hand, the proliferation of internet technologies, with, in particular, the enhanced functionalities provided by online travel intermediaries (OTAs) and metasearch engines, have made it easier for travelers to seek out, identify, and book accommodation online (Romero & Tejada, 2020; Zhao et al., 2015). Surveying a panel of 30,000 global travelers, Smith Travel Research (STR) found that 71% of travelers booked their accommodation through online channels, with the majority booking through OTAs and metasearch engines (44% of the 71%) as opposed to supplier websites (STR, 2018).

In addition, growing environmental concerns have resulted in travelers seeking out more sustainable, “green” accommodation options when planning trips (Assaker, 2020; W. G. Kim et al., 2017). With their heavy water use, generation of large amounts of solid waste, and significant percentage of CO_2_ emissions from daily operations, hotels are perceived as having a negative effect on the environment, and customers increasingly want to be seen to play their role in environmental protection and sustainability by minimizing this footprint. A survey of 50,000 users conducted by TripAdvisor in 2014 revealed that 81% of respondents were interested in hotels implementing green practices and that it would influence their choice of accommodation (TripAdvisor, 2014). Likewise, another survey by Booking.com in 2019 on 12,134 users across 12 major international markets revealed that, if presented with the option, more than two thirds (68%) were more likely to consider eco-friendly accommodation (whether they were initially looking for a sustainable stay or not) and were more likely to spend at least 5% more on their travel/accommodations to ensure it was as environmentally low impact as possible (Booking.com, 2019).

In light of these trends, and to better respond to travelers’ environmental aspirations when sourcing accommodations online, TripAdvisor has rated the green practices of hotels since 2013 (Gil-Soto et al., 2019). GreenLeaders is a free and voluntary program in which hotels can participate by highlighting six predefined green areas/green practices the hotel has in place: Energy, Water, Purchasing, Waste, Site, and Education & Innovation. Based on their rating for these practices, hotels earn one of four statuses: Bronze, Silver, Gold, or Platinum. Their GreenLeaders badge and the status level appears under the hotel’s name on their TripAdvisor page, and users can click to see a pop-up window listing their green practices (H. Lee et al., 2016). Over 11,000 properties in 66 global markets now feature the GreenLeaders badge, with GreenLeaders-accredited hotels enjoying 20% higher ratings, according to TripAdvisor (TripAdvisor, n.d.).

Given the popularity of the GreenLeaders program with both hoteliers and travelers, and continued growth in the number of travelers expected to make sustainable travel choices in the future (K. -H. Lee et al., 2019), several other online travel platforms have introduced similar systems to aid travelers in assessing green practices of hotels when booking online (Thomsen, 2018). For example, Booking.com has partnered with Green Key (an existing voluntary eco-label that can be earned by hotels at the property level) to highlight Green Key-awarded properties on its site as “sustainable” and to allow Booking.com customers to identify such properties when looking for accommodation options. Other online travel platforms, including Expedia Inc., have also started collaboration with Green Key for the same purpose, making green certification a common decision criteria on OTA websites.

And although online platforms are actively implementing green certification labels/badges, existing academic studies on the determinants of travelers’ online accommodation choice have, to date, largely failed to integrate this construct into the selection attributes investigated, focusing instead on traditional attributes such as price, location, review ratings, and brand (Dickinger & Mazanec, 2008; Eriksson & Fagerstrøm, 2018; Masiero et al., 2020; Murphy & Chen, 2014). Similarly, existing studies that have examined travelers’ accommodation choice have tended to focus on revisit intention rather than initial booking choice (see Apostolakis et al., 2020; Njite & Schaffer, 2017; Verma & Chandra, 2018). In addition, most have also largely failed to include green certification labels/badges, focusing instead on guest experience of specific green hotel practices such as recycling, energy and water conservation, and towel and linen reuse policies experienced during their stay rather than certification. This contrasts sharply with first-time bookers, who, not having visited the property and been exposed to a hotel’s green practices, cannot directly evaluate a hotel’s environmentally sustainable attributes for themselves (Millar & Baloglu, 2011), and thus are more dependent on credible third-party evidence of best practice such as green certification labels/badges to inform their decision (W. G. Kim et al., 2017). Given that existing studies have not examined the relative importance of green certification labels/badges in the initial booking process, there is a need to evaluate the role of green certification labels/badges in influencing travelers’ booking choices, both in general and in the online context (W. G. Kim et al., 2017).

To address this knowledge gap, this article uses conjoint analysis to evaluate the (relative) importance of green certification labels/badges in influencing travelers’ choices when booking a room online, comparing it to other salient hotel attributes proposed by the literature (i.e., Rating, Brand, Location, Price, Cancellation Policy, and Photos). In particular, in keeping with the conjoint analysis methodology used, each of the respondents selected for the study was presented with 12 different hotel profiles similar to what they would encounter in reality when booking online. Each profile represents various levels and characteristics of each of the abovementioned attributes. Respondents were asked to rank each hotel profile from 1 to 12, from the most to least preferred. This analysis is largely based on the theory of consumer demand (see Lancaster, 1971) which stipulates that consumers tend to choose a particular product or service based on specific attributes and attribute levels/characteristics, and select the product or service (in this case the combination of attributes and attributes levels) that maximizes their total utility based on the utility they ascribe to each individual attribute. Using the conjoint analysis technique allows us to gauge the relative utility travelers extract from each level and subsequently the relative importance of each attribute in their online booking choice (Murphy & Chen, 2014), contributing to this study’s purposes.

Furthermore, in light of the COVID-19 pandemic which is expected to modify travelers’ behavior and increase the importance of green practices (Gursoy & Chi, 2020; O’Connor & Assaker, 2021), respondents were each presented with two separate hypothetical experimental contexts, asking them to respond as if they were making a booking pre-pandemic and post-pandemic, respectively. To the authors’ knowledge, previous studies have been implemented only in the pre-COVID-19 context (Masiero et al., 2020). As such, asking respondents to rank the relative importance of these issues in both contexts allows us to identify potential changes in preferences that may result from the pandemic, as well as the relative importance of attributes (and in particular, the green certification label/badge attribute) as a consequence of COVID-19, providing further insights into pre- and post-pandemic choice behaviors. Finally, and to further gauge the influence of the green certification labels/badges on booking decisions, respondents were also asked whether they were willing to pay more for a hotel featuring a green certification label/badge.

In doing so, this article contributes to our theoretical understanding of the attributes that influence hotel selection/booking choices in the online context, in particular by advancing available models of thought regarding factors influencing the online booking process through incorporating and inspecting the relative importance of green certification labels/badges. It also examines these attributes in pre- and post-COVID-19 contexts, helping to identify whether attitudes to these attributes, particularly green certification labels/badges, have changed as a result of the pandemic. It also provides practical implications: first, for hotel operators listing their rooms on online travel platforms, allowing them to determine which attributes are most likely to influence post-COVID-19 travelers’ selection. Second, for online platforms interested in introducing green certification labels/badges, the findings provide empirical insight on whether travelers do indeed take such badges into consideration when making their hotel booking choice in the online context, helping weigh the benefits of such programs for driving future business.

The article will proceed with a review of the literature on online hotel attributes and green certifications, followed by a discussion of the study’s design, research methods and conjoint analysis, and finally the conclusion and discussion of findings.

## Literature Review

### Online Hotel Attributes

In the past decades, many scholars have conducted studies aiming at exploring consumers’ hotel selection attributes (Fu et al., 2021; Kucukusta, 2017; Millar & Baloglu, 2011; Sarwar & Azam, 2019). They contend that the heterogeneity of the hospitality product in terms of the various attributes that consumers consider when booking makes this decision a complex one. Such complexity is amplified in the online context, where the characteristics and attributes of the online travel platform can also influence travelers’ decisions (Abdullah et al., 2017).

With that in mind, reviewing existing articles on the determinants of travelers’ online hotel booking choices revealed that several have examined how the attributes of an online travel platform/booking system affect users’ decision to book a hotel (Dickinger & Mazanec, 2008). Here studies have posited on whether the quality of the booking system in terms of transaction safety and security, usefulness in terms of information depth and comparison of various hotel options, ease of use, and interactivity in terms of responsiveness and feedback were the main factors influencing online hotel booking choices and behavior (Abdullah et al., 2017; Agag and El-Masry, 2016).

Other studies have focused on identifying the relevant hotel attributes that influence booking choices in an online context using a plethora of methods including multiple regression (Kim & Kim, 2004), structural equation modeling (SEM; Hu & Yang, 2020; Sarwar & Azam, 2019), discrete choice models (Masiero et al. 2015; Fu et al., 2021), travel blog analysis (Alrawadieh & Law, 2019; Li et al., 2013), and to a lesser extent conjoint analysis (Arenoe & van der Rest, 2020; Eriksson & Fagerstrøm, 2018; Masiero et al., 2020) . While most of these methods are useful in understanding the causal effect of various factors on travelers’ online booking attitudes/intentions, only conjoint analysis is suitable for extracting information about travelers’ preferences with respect to each attribute, facilitating a better comprehension of how travelers go about making a choice, as well as determining the optimal hotel profile (based on respondents’ preferred set of attributes/attribute levels) that travelers are most likely to choose in an online context (Dickinger & Mazanec, 2008; Masiero et al., 2020; Murphy & Chen, 2014; Park et al., 2017).

More specifically, among the conjoint studies that have examined relevant hotel attributes in relation to traveler’s online hotel booking choices (and thus most relevant to the context of the present article), Dickinger and Mazanec (2008) found that (1) recommendations of friends, (2) hotel pictures, (3) reviews, and (4) price were the most important factors that influenced online hotel booking among 340 online respondents who were asked to rate 22 hotel profiles alternatives representing 4-star hotel properties in Barcelona (Dickinger & Mazanec, 2008). Eriksson and Fagerstrøm (2018), on the other hand, using a sample of 120 Finish students and a hypothetical scenario for 16 different 3* hotel profiles in a major European city, found that (a) hotel rating, (b) price, and (c) Wi-Fi reviews had the greatest influence on online booking decisions, with brand found to have a negligible influence in this case. Murphy and Chen (2014), based on a sample of 60 students enrolled in a hospitality program in Switzerland asked to rank eight hotel profiles they were likely to encounter online for their next travel destination, found that (a) review ratings and (b) number of reviews, (c) price, and (d) star ratings were key attributes in online hotel selection. Finally, Masiero et al. (2020) using a sample of 382 international tourists visiting Hong Kong and, using a hypothetical scenario of 10 different hotel profiles they could book for their current stay, found that cancellation policy had the greatest influence on consumers’ online booking decision.

Accordingly, and given that the main focus of the present article is green certification labels/badges, only the most salient attributes usually found on travel booking websites were selected for inclusion in the conjoint analysis, namely Rating (very good; average), Brand (familiar; unfamiliar), Location (centrally located; non-central), Price (high; average), Cancellation policy (free cancellation; no-refunds), and Photos (very nice pictures; average pictures). Levels for each attribute were limited to two (similar to Murphy & Chen, 2014) to limit/optimize the number of scenarios presented to respondents and avoid subject fatigue (this is further discussed in the research methods section next; also see Murphy & Chen, 2014).

### Hotel Green Certification and Practices

Recently, travelers have become increasingly environmentally conscious in their purchase of tourism and hospitality products (Assaker, 2020; Moise et al., 2021). This is no different for the lodging industry, where many travelers having realized the negative effects that hotel operations have on the environment (water consumption, solid waste generation, significant CO_2_ emissions, etc.) are increasingly opting for environmentally friendly options when they travel (Moise et al., 2018). As such, more hotels are making efforts to implement green practices and adopting sustainable guidelines in their operations with the aim to appeal more to environmentally conscious customers (W. G. Kim et al., 2017), whereas according to the signaling theory, not only can hotel environmental practices send a positive signal to customers about that hotel’s environmentally responsible, making it comparatively more attractive, but will also help customers signal and express to other customers their concern and interest in protecting the environment through their purchase of / or stay at an environmentally friendly property, thus enhancing customers’ engagement and consumption intention toward hotels that have adopted green practices or have earned green certifications (Hwang & Kim, 2021; Rahman et al., 2020). For this reason, many hotels have implemented green practices in their operations (e.g., waste reduction and recycling, energy saving, towel and linen re-usage, environmental education programs, see Assaker, 2020; Han et al., 2019), with others pursuing green certifications such as “Leadership in Energy and Environmental Design” (LEED), “Energy Star,” and “Green Seal” certification, all to appeal better to environmentally conscious travelers (K. -H. Lee et al., 2019).

While, to the authors’ knowledge, no prior studies have specifically investigated the role of green certification/badges in the hotel booking decision, previous research has clearly established the positive influence of both hotel green practices and green certifications on travelers’ attitude toward the hotel, level of satisfaction, intention to revisit, and willingness to pay more (Kang et al., 2012; Y. H. Kim et al., 2019; Nelson et al., 2021; Olya et al., 2021). However, most studies have been conducted in a post-purchase context, typically utilizing reviews of respondents who have already stayed in and experienced the hotel and its green practices (Gil-Soto et al., 2019; H. Lee et al., 2016; Song et al., 2020).

For example, H. Lee et al. (2016) performed a content analysis on environmental comments posted by reviewers on TripAdvisor with regard to 10 U.S. hotel properties that had achieved TripAdvisor’s GreenLeaders platinum status (the highest level) over a 5-year period window (2009 to 2014), finding that four of the six predefined green areas included in the GreenLeaders program (green procurement, environmental education programs, efficient energy use, recycling programs and towel reuse) were the most mentioned and positively commented-on practices in reviews posted about the selected hotels. Without directly soliciting consumers, they postulate that these green practices in particular were most likely to appeal to travelers and drive future bookings (H. Lee et al., 2016).

Similarly, Gil-Soto et al. (2019) reviewed environmental comments posted by reviewers on TripAdvisor regarding hotels in the Canary Islands and likewise were able to establish that green practices, in general, and environmental education programs, green procurement, and efficient water and energy practices, in particular, received mostly positive comments from travelers and thus were also likely to influence travelers’ positive perception and future booking.

Finally, W. G. Kim et al. (2017), using data collected from 100 hotel properties from a major U.S. hotel chain, examined the effect of customers’ rating of the selected hotel attributes of Cleanliness, Rooms’ Quality, Location, Service Quality, and Wi-Fi (with these ratings taken from Priceline, Expedia, and Hotels.com) in addition to whether the selected hotels had achieved GreenLeaders status (coded as a dummy variable and taken from TripAdvisor website) on hotel ADR, RevPar, and future rebookings (with these latter taken from the hotel chain internal data). They found that cleanliness, location, and room and service quality were all significant determinants of customers’ rebooking. And while GreenLeaders badge was not a significant determinant of rebooking, it was significantly correlated with ADR and RevPar, suggesting that travelers were willing to pay more for hotels with GreenLeaders badge status.

In addition, to the authors’ knowledge, no studies to date have examined the role of green practices and green certification specifically in the online booking context. With the growth of the web, most hotel bookings now flow through online channels and, in particular, through the major online travel platforms such as Expedia, Booking.com, making a more thorough understanding of the factors that drive conversion in this environment important. It is well established that the heterogeneous, intangible, and geographically dispersed nature of the hotel sector means that hotels cannot be inspected or experienced prior to purchase, making it difficult for potential customers to gain a true sense of a hotel property prior to booking (Bilgihan & Bujisic, 2015). With the consequences of a suboptimal choice in mind, travelers seek out as much detailed, relevant, and topical information as they can to minimize risk and make the right selection (Buhalis and O’Connor, 2005). And as marketing information provided by the seller is increasingly regarded with skepticism (Ayeh et al., 2013), credible third-party information including certification and peer reviews have taken on increased importance (Colicev et al., 2019; Litvin et al., 2008; O’Connor, 2008). However, while significant work has been carried out on establishing the role and influence of online reviews in the travel booking process (see, for example, Chevalier & Mayzlin, 2006; Öğüt & Onur 2012; Nieto-Garcia et al., 2019; Sparks & Browning, 2011), few studies have specifically considered how independent badges/certification in general and, in particular, green certifications, influence travelers’ choice when booking a hotel through online channels (Mariani & Borghi, 2021).

Thus, while existing research establishes the importance of green practices and certification in the post-purchase and rebooking context, the relative importance of these issues in influencing the initial booking remains to be established, both in general and in the online booking context. Accordingly, there is a need to further investigate the relative importance role of green certification labels/badges on travelers’ online booking decisions. As such, Tripadvisor’s GreenLeaders badge attribute (whether the hotel has earned the GreenLeaders status or not) is used alongside the previously discussed attributes of Rating, Brand, Location, Price, Cancellation Policy, and Photos in the present study to examine the relative importance of attributes in terms of the hotel choice decision in the online booking context, as well as travelers willingness to pay more for certified properties. In addition, given that this study was conducted in the midst of the COVID-19 pandemic, and in light of the many recent studies on the pandemic’s effect on travelers’ behavior, heightened environmental concerns, and willingness to travel more sustainably (Jian et al., 2020; O’Connor & Assaker, 2021), two experimental contexts/scenarios (pre- and post-COVID-19) were used to investigate potential differences in the relative importance of the selected hotel attributes (and in particular green certification labels/badges) on travelers’ online hotel selection criteria. The study design and conjoint analysis are further explained in the next section.

## Research Methods

### Sample, Study Designs, and Attributes Measurement

The sample for this study was collected during the last week of April 2020 by a leading U.K.-based market research company from a country panel representative of the U.S. population. A total of 672 respondents were randomly selected from the U.S. panel; of those 672 selected respondents, only those who stayed overnight at a midscale/upmarket hotel for leisure purposes in the previous 12 months and had also used TripAdvisor at least once in the past 12 months in the context of travel planning were retained. This was to ensure that all respondents were familiar with TripAdvisor as well as other salient hotel attributes usually found on online travel platforms, and that selected respondents had recently been through the experience of searching for a hotel as the same context would be replicated in the article’s data collection process. Moreover, only midscale/upmarket were considered for this study as they are more likely to apply and communicate green practices compared with lower-scale properties (Moise et al., 2021). In addition, such properties are more affordable than luxury hotels, helping to ensure adequate participation rates (Assaker, 2020). As a result, 306 respondents (out of the 672 initially selected respondents) were allowed to participate in the study.

Respondents were then exposed to two distinct online scenarios. In the first, they were asked to assume that they were planning to take a two-night trip to a popular tourist destination prior to the current COVID-19 pandemic and search TripAdvisor to find a hotel to book. Then they were presented with a list of 12 hotel options/profiles that closely simulated the results they would receive in real life from TripAdvisor and asked to rank the presented options from the most favorable (in this case, Rank 1) to the least favorable (in this case, Rank 12). In the second scenario, respondents were subject to the same study design and hotel options/profiles except they were asked to imagine that their trip would take place once the COVID-19 pandemic had become under control.

Seven attributes were used to generate the 12 hotel options/profiles used in both scenarios, which are the GreenLeaders badge, Rating, Brand, Location, Price, Cancellation policy, and Photos and with each attribute consisting of two levels as discussed earlier and as shown in Table 1. Also, it is worth noting that when respondents were presented with the set of hotel profiles, they were also instructed that free Wi-Fi, breakfast, and parking were included in the price of all options presented to them. In addition, all options/profiles offer the same type/level of basic amenities (bar/lounge, restaurant, fitness center, 24-hr security, etc.) to control for the effect of these latter variables on respondents’ decision when ranking the 12 hotel profiles provided based on their perception of the seven attributes being examined in this study (Arenoe & van der Rest, 2020).

**Table 1.** Selected Online Hotel Attributes and Their Respective Levels.

Attributes	No. of Levels	Attribute Level 1	Attribute Level 2
Rating	2	Very good	Average
Brand	2	Familiar	Unfamiliar
GreenLeaders badge	2	Yes	No
Location	2	Centrally located	Non-central
Price	2	Average	High
Cancellation Policy	2	Free cancellation	No refund
Photos	2	Very nice pictures	Average pictures

### Profiles Selection and Data Analysis

In deciding upon the number of profiles to be presented to respondents, and bearing in mind that it would be impossible to ask respondents to rank all possible profiles containing all levels of all attributes, we used the well-established rule of thumb suggesting that the number of profiles should be 1.5 times the number of parameters to be estimated, with the latter determined by the formula *n* (*k* − 1) + 1, where *n* = the number of attributes, and *k* = the number of levels for each attribute (Verma & Chandra, 2018). With seven attributes having two levels each in this study, there would be eight parameters, 7(2 − 1) + 1 = 8, and thus, 12 hotel options/profiles were presented to each participant as mentioned earlier. Using conjoint analysis, this allowed us to extract the desired information about respondents’ preferences, without causing respondent fatigue and irregular data (Eriksson & Fagerstrøm, 2018).

Moreover, in choosing how to group the different levels within each profile, we used SPSS Orthogonal Design procedure (ORTHOPLAN), also known as orthogonal arrays, to ensure that there would be no correlation among the different levels of each attribute and that each level of each attribute appeared the same number of times throughout the 12 proposed hotel options/profiles (see Kuzmanovic et al., 2011). This allowed us to link the profile with the highest ranking to one specific attribute level, and to gauge respondent preferences for all attribute levels used accordingly to define the 12 hotel profiles. Table 2 details the full list of the 12 hotel profiles used, along with their respective attribute levels from the Orthogonal Design Procedure.

**Table 2.** Full List of Hotel Profiles Based on SPSS Orthogonal Design procedure (ORTHOPLAN).

Hotel Profiles	Overall Rating	Brand	GreenLeaders Badge	Location	Price	Cancellation Policy	Photos	Ranking (1 = Most preferred to 12 = Least preferred)
1	Very good	Familiar	Yes	Centrally located	High	Free cancellation	Very nice pictures	
2	Very good	Unfamiliar	Yes	Non-central	High	No refund	Very nice pictures	
3	Very good	Familiar	No	Centrally located	Average	Free cancellation	Average pictures	
4	Average	Unfamiliar	No	Centrally located	High	No refund	Average pictures	
5	Average	Unfamiliar	No	Centrally located	High	Free cancellation	Very nice pictures	
6	Very good	Unfamiliar	No	Non-central	Average	Free cancellation	Very nice pictures	
7	Average	Familiar	Yes	Non-central	High	Free cancellation	Average pictures	
8	Average	Familiar	Yes	Centrally located	Average	No refund	Very nice pictures	
9	Very good	Familiar	No	Non-central	High	No refund	Average pictures	
10	Average	Unfamiliar	Yes	Non-central	Average	Free cancellation	Average pictures	
11	Average	Familiar	No	Non-central	Average	No refund	Very nice pictures	
12	Very Good	Unfamiliar	Yes	Centrally located	Average	No refund	Average pictures	

Full-profile or rating-based conjoint was used in this case because it allows the extraction of individual preferences (also called partworth scores or utilities) for each respondent with respect to each of the selected attribute levels (Millar & Baloglu, 2011). This is done through a series of multiple regression procedures performed on each respondent’s stated ranks, where the utilities of each respondent is similar to the coefficients in a multiple regression such that each utility or partworth score represents the respondent “desirability” or “preference” for a particular attribute level (Hair et al., 2010). Individual utilities/partworth scores are then used to produce the relative importance of each attribute for every respondent, and most importantly, those individual results are then ultimately averaged to accurately compute the relative importance of the different attribute levels/attributes, which are in turn used to interpret the results from the conjoint procedure (Orme, 2013). As such, full-profile or rating-based conjoint analysis typically leads to more accurate results at the aggregate (sample) level by allowing researchers to extract more detailed levels of information, as opposed to Choice-Based and other types of conjoint procedures (Hair et al., 2010). The only constraint to using full-profile conjoint is that the number of attributes should not exceed 10, with no more than two to three levels per attribute. Exceeding these levels would lead to a large number of complex profiles and the need for respondents to resort to simplification strategies to rank profiles, in addition to fatigue, leading to irregularities and noisy data (Orme, 2013). With seven attributes used in the present study and two levels per attribute, full-profile conjoint analysis is thus suitable to analyze our data with the results, presented next.

## Analysis and Results

Before running conjoint analysis on our selected sample of 306 responses, we checked the data for irregularities or unrealistic responses. We found and deleted 36 cases where respondents ranked every hotel profile the same way as they were presented to them in the list either in ascending (1 to 12) or descending order (12 to 1), suggesting that respondents did not take the time go through the profiles and assign ranks according to their respective desirability or preference. This is a common phenomenon in conjoint studies and was expected in our study where respondents were asked to respond to two scenarios (pre- and post-COVID-19), thus increasing their chances of fatigue and affecting their willingness to properly complete the exercise. As a result, the sample was reduced to 270 valid responses, which is still greater than the threshold of 200 respondents recommended by Hair et al. (2010) for full-profile or rating-based conjoint analysis. The conjoint procedure was subsequently performed on those 270 responses.

### Sample Demographics

Of the 270 respondents, 59% were male, with half of the respondents (50%) under 40. The largest age categories were 18 to 40 years (50%), followed by 41 to 65 years (44.8%), and finally 66+ years (5.2%). Of the 270 participants, 25% had completed primary or high school (including vocational diplomas), 30% had a bachelor’s degree, and 45% had post-graduate qualifications. The income of the respondents was slightly skewed toward the upper quartile of household income brackets, with 57% earning more than USD 80,000 a year, 30% between USD 40,000 and USD 80,000, and 13% earning less than USD 40,000. This result is expected in most tourism studies, given the high-end nature of the hospitality and tourism product (Assaker, 2020) and the requirement to have stayed at a midscale/upscale hotel in the past year to participate. Finally, more than two third (76%) of respondents were married as opposed to 17% single and 7% divorced/separated or widowed. Overall, the sample demographics show that the sample is well spread out and closely representative of the concerned population (see The United States Census Bureau, 2020) with the exception of income and marital status, which are more specific to the context of the present study in this case.

### Conjoint Results

The goodness of fit of the conjoint model was upheld where the results of the Pearson’s R and Kendall’s Tau revealed a strong positive correlation between the observed and estimated ranks in pre-COVID-19 (Pearson’s R = 0.941, *p* < .000; Kendall’s Tau = 0.727, *p* < .000), and post-COVID-19 (Pearson’s R = 0.964, *p* < .000; Kendall’s Tau = 0.758, *p* < .000) scenarios from all 270 respondents, with both statistics significant and greater than the 0.6 threshold proposed by Hair et al. (2010), suggesting the validity of the data sample in predicting respondents’ online hotel choices in both scenarios. Moreover, all Pearson’s R from each individual respondent were greater than the threshold of 0.5 proposed by Moskowitz et al. (2002), further indicating that the proposed conjoint model is a good predictor for each individual respondent and that there was no need to eliminate cases on the basis of validity/prediction criteria (see Millar & Baloglu, 2011).

In addition, Table 3 shows the relative importance of each of the seven attributes on respondents’ online hotel selection/booking choice according to the ranking of their likelihood of booking the 12 hotel profiles presented. In particular, in both scenarios (pre- and post-COVID-19), cancellation policy was the most important attribute exerting an influence of 17.95% and 18.54% on respondents’ online hotel selection/decision, respectively. In the pre-COVID-19 scenario, hotel rating was the second most important attribute (17.71%), followed by price (16.06%) and location (14.95%), while in the post-COVID 19 scenario, price (16.45%) was the second most important attribute, followed by rating (16.27%) and location (15.26%), suggesting respondents were more sensitive to price when selecting their hotel room online post-COVID-19. Moreover, the remaining three attributes, that is, photos, brand, and GreenLeaders badge, were found to have the least influence on respondents’ online hotel selection across both scenarios, with GreenLeaders badge, the main variable of interest in this case, having only a 10.69% and 10.61% influence on respondents’ selection pre- and post-COVID-19, respectively.

**Table 3.** Relative Attribute Importance Scores: Pre- and Post-COVID-19.

Attributes	Pre-COVID-19		Post-COVID-19	
	Importance Score^a^	Rank	Importance Score^a^	Rank
Rating	17.71	2	16.27	3
Brand	11.18	6	10.98	6
GreenLeaders badge	10.69	7	10.61	7
Location	14.95	4	15.26	4
Price	16.06	3	16.45	2
Cancellation policy	17.95	1	18.54	1
Photos	11.46	5	11.88	5

a. Averaged importance score from all 270 respondents.

Table 4 also shows the utilities that respondents associate with each attribute. In this case, the levels with the highest positive partworth or utility scores are the ones most preferred by respondents (Millar & Baloglu, 2011). In particular, across both scenarios (pre- and post-COVID-19), respondents preferred properties that offer free cancellation (utility is equal to .798 and .857, respectively), had a very good rating (.712 and .682, respectively), were averagely priced (.156 and .075, respectively), and were centrally located (.557 and .474, respectively). Moreover, respondents also exhibited a higher preference for properties with attractive pictures, those owned/operated by familiar brands, and those that have the GreenLeaders badge (see Table 4), despite the lower relative importance of these latter three attributes in respondents’ respective online hotel selection/decision.

**Table 4.** Partworth Utility Scores for Each Attribute Level: Pre- and Post-COVID-19.

	Attribute Levels	Pre-COVID-19		Post-COVID-19	
		Utility Estimate^a^	*SE*	Utility Estimate^a^	*SE*
Rating	Very good	.712	.072	.682	.067
	Average	–.712	.072	–.682	.067
Brand	Familiar	.198	.052	.217	.054
	Unfamiliar	–.198	.052	–.217	.054
GreenLeaders badge	Yes	.014	.051	.056	.052
	No	–.014	.051	–.056	.052
Location	Centrally located	.557	.063	.474	.064
	Non-central	–.557	.063	–.474	.064
Price	Average	.156	.074	.075	.075
	High	–.156	.074	–.075	.075
Cancellation Policy	Free cancellation	.798	.067	.857	.069
	No refund	–.798	.067	–.857	.069
Photos	Very nice pictures	.344	.051	.356	.052
	Average pictures	–.344	.051	–.356	.052
(Constant)		6.500	.000	6.500	.000

a. Averaged utility score from all 270 respondents.

Finally, the above preferred levels taken together represent the ideal combination of online hotel selection attributes that yields the optimal hotel profile (i.e., the ideal hotel profile that generates the highest total utility and that respondents are most likely to book), with total utility in this case computed by summing the positive partworth/utility scores of each level (Verma & Chandra, 2018). By adding all positive utility scores along with the constant from Table 4, we obtained a total utility of 9.278 and 9.217 for the pre- and post-COVID-19 scenarios, respectively. According to the maximum utility probability (also known as first choice method, see Hair et al., 2010), this would result in 55.2% and 53.4% of the total 270 respondents choosing this hotel over all other possible profiles.

### Price Sensitivity

Although the GreenLeaders badge, compared with other examined attributes, was found to have a relatively low relative importance in influencing online hotel selection/booking choice, results from Table 5 show that 44.7% and 44.1% of the total 270 respondents in the pre- and post-COVID-19 scenarios were willing to pay more for a hotel that has obtained the GreenLeaders badge on TripAdvisor as opposed to a hotel that has not. However, more people were also willing to pay less for such a hotel in the post COVID-19 scenario (*N* = 28 or 10.4%) compared with the pre-COVID-19 (*N* = 13 or 4.8%), with these differences in proportions further upheld at the population level given that results from inferential statistics performed on the two scenario proportions show almost no overlap between lower and upper bound proportions at the 95% confidence interval (see Table 5). This further supports the higher importance of price in general and the larger sensitivity of some respondents to price in the post-COVID-19 online hotel selection scenario.

**Table 5.** Price Sensitivity Toward GreenLeaders Badge: Pre- and Post-COVID-19.

Would You Be Willing to Pay More, Less, or the Same for a Hotel That Has Achieved TripAdvisor’s GreenLeaders Certification?				
Pre-COVID-19				
	Frequency	%	95% Confidence Interval	
			Lower	Upper
More	128	47.4	41.4	53.4
Less	13	4.8	2.3	7.4
The same	129	47.8	41.8	53.8
Total	270	100.0		
Post-COVID-19				
	Frequency	%	95% Confidence Interval	
			Lower	Upper
More	119	44.1	38.1	50.0
Less	28	10.4	6.7	14.0
The same	123	45.6	39.6	51.5
Total	270	100.0		

## Conclusion and Discussion

In light of today’s travelers’ growing environmental concerns and preference for sustainable alternatives when they travel, this study aimed at investigating the relative importance of the green certification labels/badges in travelers’ online hotel selection/booking choices. By performing conjoint analysis on seven salient hotel attributes commonly present on online booking platforms (Rating, Brand, Location, Price, Cancellation Policy, Photos and green certification labels/badges), the study aimed to enhance our understanding of travelers’ choices when booking a hotel room online, as well as the relative importance of each attribute in influencing consumers in both the pre- and post-COVID-19 booking scenarios. The results provide both theoretical and practical contributions.

From a theoretical perspective, the results help extend our understanding of the application of the theory of consumer demand in the online hotel booking context, where existing theory stipulates that travelers choose a particular product or service (a hotel room in this case) based on the utility they derive from the physical properties or specific attributes of that product/service. It is these individual utilities, when summed up, that help determine travelers’ total utility from the product/service and subsequently their selection (Lancaster, 1971; Millar & Baloglu, 2011). While previous scholars have examined the importance/role of certain salient online hotel selection attributes usually found on online travel platforms in their respective studies, to the author(s) knowledge none have incorporated green certification labels/badges into their respective models. As such, this present study helps fill this gap by incorporating and exploring the relative importance of the GreenLeaders badge in the online hotel selection process, together with the previously identified attributes from existing studies. Findings show that, contrary to expectations and despite media hype, green certifications/badges were found to only play a minor role in travelers’ online hotel selection, that is, exerting a 10.69% and 10.61% influence on respondents’ selection in both pre- and post-COVID-19 scenarios, respectively. However, travelers did show a preference for hotels that display a green certification label/badge as opposed to those who do not. Thus, despite growing consumer concerns about sustainability and the negative environment effects of mainstream travel, such badges were found not to be as important relative to other attributes such as cancellation policy, rating, price, and location, which together accounted for around 67% of travelers’ decisions across the two examined scenarios (pre- and post-COVID-19).

While these results partially support findings from previous articles that highlighted the importance of hotel green practices and green certifications for travelers’ satisfaction and revisit intention in an offline context (Kang et al., 2012; Y. H. Kim et al., 2019; Nelson et al., 2021), as well as other articles conducted that show that a hotel’s green practices as reflected through guests’ review positively influence customers/travelers perception (Gil-Soto et al., 2019; H. Lee et al., 2016; Song et al., 2020), the present study clarifies that green certifications, and green certification labels/badges specifically, are not as important and significant in influencing travelers’ choice as one might expect in the online hotel booking context. Although beyond the scope of this study, we speculate that this could be explained by cognitive dissonance theory, which involves divergences between travelers’ desire (what they feel they should do) and actual behavior (what they actually do) when it comes to environmental choices (see Kasim, 2004; W. G. Kim et al., 2017). While many travelers profess to be concerned about the environment, this does not necessarily mean that they will actively consider environmental attributes in their hotel selection process. This runs counter to the findings of an abundance of articles (e.g., Jian et al., 2020; O’Connor & Assaker, 2021) that have advanced that travelers’ growing consideration for today’s environment, in particular as a result of the recent pandemic which has heightened people’s worries about environmental, health, and safety concerns, would lead to consumers behaving more sustainably, particularly when they travel (Gursoy & Chi, 2020).

In addition to the relative importance of green certification labels/badges in the context of online hotel selection, the present study also examined and reconfirmed the importance of the salient hotel attributes of cancellation policy, rating, price, and location in travelers’ online hotel selection, further aligning with previous studies on the topic. However, prior studies were conducted pre-COVID-19, and as such by examining the relative importance of online hotel selection attributes in both a pre- and post-COVID-19 scenario, this present study revealed that price is likely to play a more important role in the post-pandemic travelers’ online hotel selection. Again, this stands in opposition to several recent studies published that have argued that safety and security issues prompted by the pandemic, as well as growing environmental concerns, would make travelers more willing to pay more to minimize these two concerns (see Gursoy & Chi, 2020; O’Connor & Assaker, 2021). While findings from the present study suggest the opposite, it is worth noting that a significant and similar number of respondents were willing to pay more for a hotel with GreenLeaders badge (47.4% and 44.1% for pre- and post-COVID-19, respectively). This suggests that while travelers fail to consider the GreenLeaders badge a key attribute in their online hotel selection decision, they are willing to pay more for their selected hotel if it happened to be certified. These results support those of other recent studies, for instance, W. G. Kim et al. (2017), which found that while green certification was not a significant determinant of travelers’ online rebooking, it was significantly correlated to ADR and RevPar, which could be explained by the fact that while customers might not be willing to prioritize hotel green practices or certification at the expense of their comfort (in terms of the other hotel attributes that they desire to have), if, in addition to their desired attributes (comfort), the hotel also has green certification they might be willing to alter their behavior to make some economic sacrifices and play their part in protecting and conserving the environment (O’Connor & Assaker, 2021). This latter behavior may align with social identity theory, whereby customers/travelers might be willing to undertake additional actions to identify with additional behavior that makes them look/appear better to society as a whole (see Kang et al., 2012).

From a practical perspective, the present study’s results can help hotel operators that list their rooms on online travel platforms to understand which attributes are most likely to influence post-COVID-19 travelers selection. For instance, the findings strongly suggests that hoteliers should offer free cancellation as this was found to exert the greatest level of desirability/utility for potential travelers/customers in both the pre- and post-COVID-19 scenarios. Travel involves uncertainty; as such, travelers would feel more secure in making a booking knowing they have the option to cancel in case of emergency. Hoteliers should also list their rooms at convenient prices as properties with average (as opposed to high) prices were found to generate higher-level utility for potential travelers. While price was important pre-COVID-19, the relative importance of price in travelers’ choice was found to be even greater in the post-COVID-19 scenario, suggesting that hotel operators should revisit their pricing strategies if they wish to entice post-COVID-19 travelers and optimize the likelihood of online room bookings.

Finally, and most importantly, hotel operators should seek to achieve green certification labels/badges. Although these only marginally increase the desirability of a property, they help operators achieve a greater price margin by allowing hotels to charge a higher price. At least 40% of respondents in the present study stating that they would be willing to pay more for a hotel that has achieved TripAdvisor’s GreenLeaders certification. That being said, as discussed earlier, hotels need to be cautious as post-COVID-19 travelers seem to be more sensitive to price, with results further showing a greater percentage of travelers willing to pay only the same or even less for hotels featuring green certification, Nevertheless, achieving green certification can still be beneficial for hotels. Even if they cannot benefit in terms of bookings and prices, engaging in the process allows them to set up more efficient operations (e.g., less energy and water consumption, recycling, etc.), which would help save money and boost their reputation.

Results from this study also provide useful practical insights to online travel platforms as to the importance of incorporating a green certification label/badge on their hotel listings. In particular, while TripAdvisor’s program has received much attention since its launch in 2013, and while the platform has often touted the importance of the GreenLeaders badge for hotels to appeal to today’s more environmentally concerned consumers, there is little objective evidence to support these claims. Our findings suggest that the green certification labels/badges only play a marginal role in influencing consumers/travelers hotel booking decision in the online context. This could be due to discord between travelers’ environmental desires in general and actual behavior in particular, with their desire for comfort and other salient hotel attributes, rather than environmental concerns, taking on more importance when it comes to booking a hotel room. However, it could also be due to the self-declared nature of label/badge put in place by TripAdvisor, or by the company not effectively promoting or educating users about the program, resulting in customers failing to assign the proper level of utility and desirability to the GreenLeaders badge/status’ attribute when booking a hotel on online platforms. With other online platforms introducing their own initiative to allow travelers to assess the sustainability efforts of a hotel, the findings of this present study support the need for future schemes to be based on clear, measurable, and objective environmental scales that can be easily understood by consumers. In addition, travelers need to be educated about these initiatives so they understand what they entail in terms of positive environmental implications and sustainability. Perhaps one way to reach as many customers as possible would be by building partnership between as many competing online travel platforms as possible so that the same label/badge could be used industry wide. This would help travelers develop an enhanced awareness of the importance and the meaning of green certification labels/badges and perhaps assign additional weight to these initiatives when making their online hotel booking decisions.

## Limitations and Future Research

This study is not without limitations. First, to ensure homogeneity in expectations among respondents, only travelers who had stayed at 3 and 4* properties were surveyed for this the conjoint study. Thus, our findings might be specific to this type of travelers and might not be generalizable to other market segments. As such, there is a need for further studies to examine how people’s preferences for the attribute levels selected in this study, as well as the attributes relative importance, vary across travelers’ type (e.g., midscale, luxury, and budget). Second, data were collected only from U.S. consumers, and results might be specific to this market. Future studies could use a similar methodology to collect cross-national data to examine differences in preferences and comparative choice patterns across cultures/nationalities. Third, this study investigated seven attributes, with two levels each; future studies could add more attributes and attribute levels to the conjoint model to better understand the traveler’s online hotel selection processes. This would, however, require the use of more advanced conjoint analysis (e.g., Adaptive Choice-Based Conjoint), requiring more advanced software to allow for adaptation of the profiles shown to each respondent and for analysis of a larger amount of data (attributes/attribute levels), but would help generate more detailed results and insights on travelers’ online hotel selection. Finally, the underlying cause of the major difference between consumers’ stated preferences and actual booking actions remains unclear. Further research, perhaps using a mixed methods approach involving experimentation and follow-up interviews, could be used to clarify this important issue and further add to our understanding of the attributes that influence hotel booking behavior in the online context.
